# nNOS-positive minor-branches of the dorsal penile nerves is associated with erectile function in the bilateral cavernous injury model of rats

**DOI:** 10.1038/s41598-017-18988-2

**Published:** 2018-01-17

**Authors:** Yen-Lin Chen, Ting-Ting Chao, Yi-No Wu, Meng-Chuan Chen, Ying-Hung Lin, Chun-Hou Liao, Chien-Chih Wu, Kuo-Chiang Chen, Shang-Shing P. Chou, Han-Sun Chiang

**Affiliations:** 1 Department of Pathology, Cardinal Tien Hospital, New Taipei, Taiwan; 2 Department of Chemistry, Fu-Jen Catholic University, New Taipei, Taiwan; 3 Graduate Institute of Biomedical and Pharmaceutical Science, Fu-Jen Catholic University, New Taipei, Taiwan; 4 Medical Research Center, Cardinal Tien Hospital, New Taipei, Taiwan; 5 School of Medicine, Fu-Jen Catholic University, New Taipei, Taiwan; 6 Graduate Institute of Medical Sciences, National Defense Medical Center, Taipei, Taiwan; 7 Division of Urology, Department of Surgery, Cardinal Tien Hospital, New Taipei, Taiwan; 8 Department of Urology, School of Medicine, Taipei Medical University Hospital, Taipei, Taiwan; 9 Department of Urology, Cathay General Hospital, Taipei, Taiwan

## Abstract

The changes in neuronal nitric oxide synthases (nNOS) in the dorsal penile nerves (DPNs) are consistent with cavernous nerve (CN) injury in rat models. However, the anatomical relationship and morphological changes between the minor branches of the DPNs and the CNs after injury have never been clearly explored. There were forty 12 week old male Sprague-Dawley rats receiving bilateral cavernous nerve injury (BCNI). Erectile function of intracavernous pressure and mean arterial pressure were measured. The histology and ultrastructure with H&E stain, Masson’s trichrome stain and immunohistochemical stains were applied on the examination of CNs and DPNs. We demonstrated communicating nerve branches between the DPNs and the CNs in rats. The greatest damage and lowest erectile function were seen in the 14^th^ day and partially recovered in the 28^th^ day after BCNI. The nNOS positive DPN minor branches’ number was significantly correlated with erectile function. The sub-analysis of the number of nNOS positive DPN minor branches also matched with the time course of the erectile function after BCNI. We suggest the regeneration of the DPNs minor branches would ameliorate the erectile function in BCNI rats.

## Introduction

The incidence of prostate cancer has persistently increased and is still increasing globally^1,2^. Radical prostatectomy (RP) surgery is the gold standard for the treatment of localized prostate cancer. Although the surgical technique has become more advanced with less morbidity, the major complication of erectile dysfunction (ED) remains inevitable^3–8^. Erectile function can be recovered months or years after surgery, but the erectile capacity often remains poor. In the literature review performed by Burnett *et al*.^9^, the authors reported that the ranges of rates for complete ED, partial ED, and intact ED were 26% to 100%, 16% to 48%, and 9% to 86%, respectively, after RP surgery.

To explore the mechanism and treatment of ED after RP surgery, various methods of inducing injury to the cavernous nerves (CNs) have been developed to mimic the damage associated with RP. They included CN resection^10–13^, transection^14,15^, crushing^16–19^ and dissection^20,21^. In addition to decreased intracavernous pressure (ICP) with evidence of corpus cavernosum fibrosis, changes in neuronal nitric oxide synthases (nNOS) in the dorsal penile nerves (DPNs) are major criteria to diagnose ED caused by CN injury^22–26^. The DPNs are crucial for normal erectile and ejaculatory function, and they serve as an afferent branch of the bulbocavernous reflex^27,28^. The DPNs originate from the pudendal nerve at the inferior part of the greater sciatic foramen, and they accompany the internal pudendal artery or vein or other branches of the pudendal nerve into the pudendal canal on the lateral wall of the ischiorectal fossa^29^. At the ventrocaudal margin of the pubis, the DPNs run in close proximity to the insertion of the crus of the penis and further between the crus and the ventral surface of the pubic body to the penile dorsum to supply the body and glans of the penis^28^.

However, a frequently encountered question by researchers who study the bilateral cavernous nerve injury (BCNI) model is whether the nNOS changes in the DPNs of rats could represent CN injury. In general, the CNs belong to the parasympathetic nerves, whereas the DPNs are somatic nerves. In a rat experiment model, Podlasek *et al*.^24^ proved that the changes in nNOS in the DPNs were consistent with CN injury. Moreover, the existence of communicating nerve branches connecting the DPNs and the CNs in human adult and infant cadavers has also been reported^30–34^. However, the anatomical relationships and morphological changes between the DPNs and the CNs after BCNI have not been clearly demonstrated in rats. In addition, changes in the minor branches of the DPNs have never been described before. In this study, we attempted to explore them in detail.

## Materials and Methods

### Experimental Animals

Forty 12 week old male Sprague-Dawley rats (weight, 450–600 g) were used in this study. There were 8 rats in each group. All of the animals were supplied by BioLasco Taiwan Co., Ltd. (Taipei, Taiwan), and the study was approved by the Fu Jen Catholic University Animal Care and Use Committee (IACUC approval NO: A10320). All of the study procedures and methods were performed in accordance with the approved guidelines.

### Experimental Design

The animals were randomly assigned to 5 groups, including a sham group and four groups of rats on the 7^th^ day, 14^th^ day, 21^th^ day and 28^th^ day after BCNI. At the end of the 28^th^ day in the sham group and each specific day in the BCNI groups, erectile response was measured. In addition, histology by H&E stain, Masson’s trichrome stain, immunohistochemical stain and transmission electron microscope observation of the middle piece of penile tissue was performed. Finally, gross dissection of the communicating branches between DPNs and CNs was performed on the sham group rats.

### Surgical Procedures

For the surgical procedure, the animals were first anesthetized with an intraperitoneal injection of sodium pentobarbital (40 mg/kg). After the abdomen was shaved and cleaned with an iodine-based solution, a lower midline abdominal incision was made. The prostate gland was exposed, and the posterolateral CNs and the major pelvic ganglion were identified. Apart from the sham group, all of the rats were subjected to BCNI. In the sham group, no further surgical procedures were performed, and the abdomen was closed. In the study groups, the CNs were isolated and crushed for 2 min per side at 5 mm from their origin in the MPG, using a hemostat clamp (Roboz Surgical Instrument Co. Inc., Gaithersburg, MD, USA).

### Measurement of Erectile Responses and Arterial Blood Pressure

The CNs were exposed and isolated via a repeat midline abdominal incision, and the crura of the penis were identified. A 24-G needle containing 50 U/mL heparin solution was inserted into the right penile crus and was connected to polyethylene-50 tubing for ICP measurement with an MP36 pressure transducer (Biopac Systems Inc., Goleta, CA, USA) and BSL software, version 3.7.3. The CNs were stimulated using a bipolar stainless steel electrode. Monophasic rectangular pulses were generated by a computer with a DS3 constant current isolated stimulator (AutoMate Scientific Inc., CA, USA). The stimulus parameters included a 7.5 mA amplitude, 20 Hz frequency, 0.2 ms pulse width, and 60 s duration. Arterial blood pressure was assessed concurrently with ICP. A real-time response of the erectile tissue was determined based on the maximal ICP, the minimum ICP, the changes in ICP (∆ICP), the area under the ICP curve and the mean arterial blood pressure (MAP).

### Histology, Masson’s Trichrome Stain and Immunohistochemistry

The constructed paraffin-embedded tissue blocks were cut in 5-μm-thick sections to perform H&E staining, Masson’s trichrome staining and immunohistochemical staining. Immunohistochemical staining was performed using the Ventana BenchMark XT automated stainer (Ventana, Tucson, AZ). The primary antibodies of neurofilament (1:50, MS-359-S1; Thermo Fisher Scientific, Cheshire, UK), tyrosine hydroxylase (1:100, Abcam, Cambridge, UK), nNOS (1:100, sc648; Santa Cruz Biotechnology, Santa Cruz, CA, USA) and Ki-67 (1:100, Abcam, Cambridge, UK) were performed. A Ventana UltraView DAB detection kit was applied, and the slides were counterstained with hematoxylin, dehydrated, and mounted. The determined criterion for the main and minor branches of the DPNs were the sizes of the nerve branches. In fact, the sizes of the main branches of the DPNs were similar to those of the dorsal artery. Therefore, if the nerve branches were smaller than the dorsal artery at the dorsal side of the penis between the tunica albuginea and the Buck’s fascia, it was determined to a minor branch of the DPNs.

### Transmission Electron Microscopy

Segments (2 mm each) of nerve tissue from the middle portion of the penis were obtained from each of the rats. All of the segments were cut into small pieces, fixed in 2.5% phosphate-buffered glutaraldehyde (0.1 M, pH 7.2) overnight and postfixed in 1% phosphate-buffered osmium tetroxide (0.1 M, pH 7.2). The pieces were then dehydrated through graded concentrations of ethanol and embedded in Epon-812. One micron semi-thin sections were stained with toluidine blue. Ultrathin sections from selected blocks were stained with uranyl acetate and lead citrate. All of the sections were analyzed with a JEOL JEM-1400 transmission electron microscope (JEOL, Japan).

### Statistical Analysis

All of the statistical analyses were performed using SPSS software, version 18.0 (SPSS Inc., Chicago, IL). Data are presented as the mean ± standard deviation. To compare the differences in each component among groups, one-way ANOVA was used. The correlations between the numbers of nerve branches and the erectile function by ∆ICP were evaluated using Pearson’s correlation analysis. A *p* value (two-sided) < 0.05 was considered to be significant.

## Results

**Figure 1:**
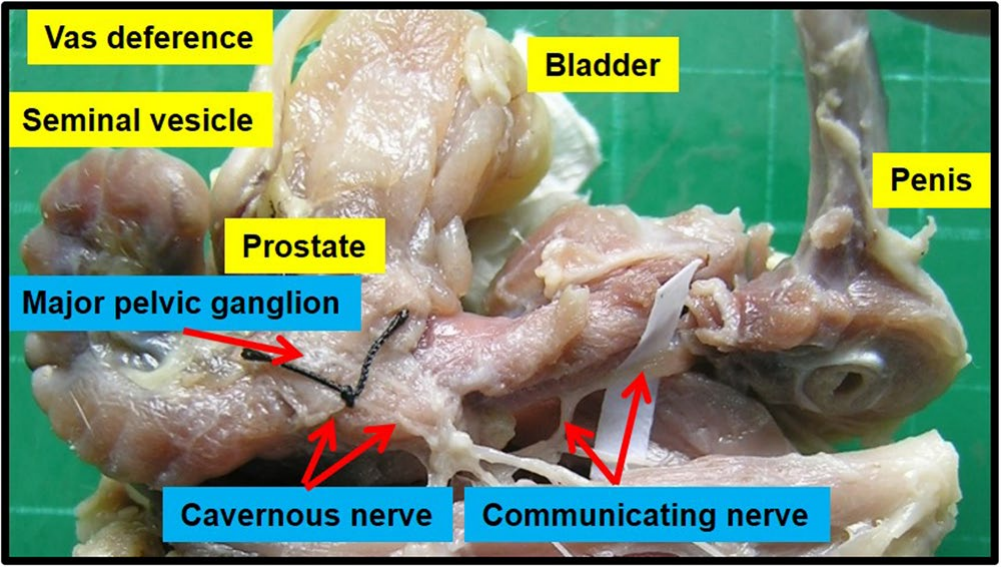
Gross dissection to demonstrate the dorsal penile nerves connected with the cavernous nerves through communicating nerve branches in rats. Gross dissection demonstrated the dorsal penile nerves connect with the cavernous nerves through the communicating nerve branches in rats. The cavernous nerve was confirmed by increasing intracavernosal pressure when the rat was alive and making a suture mark on it. There were two ganglions in the distribution of the cavernous nerve and pelvic muscles.

**Figure 2:**
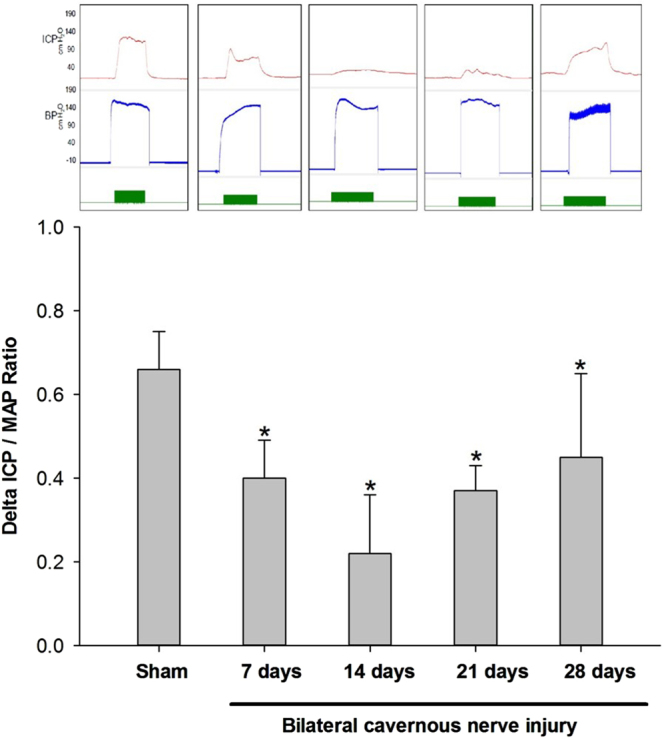
Erectile function at different days after bilateral cavernous nerve injury. According to the time course of erectile function on different days after BCNI, the erectile function was the worst on the 14^th^ day after BCNI. Subsequently, the erectile function gradually increased over the following 14 days. BCNI = Bilateral cavernous nerve injury. N = 40; Asterisk: p < 0.05.

**Figure 3:**
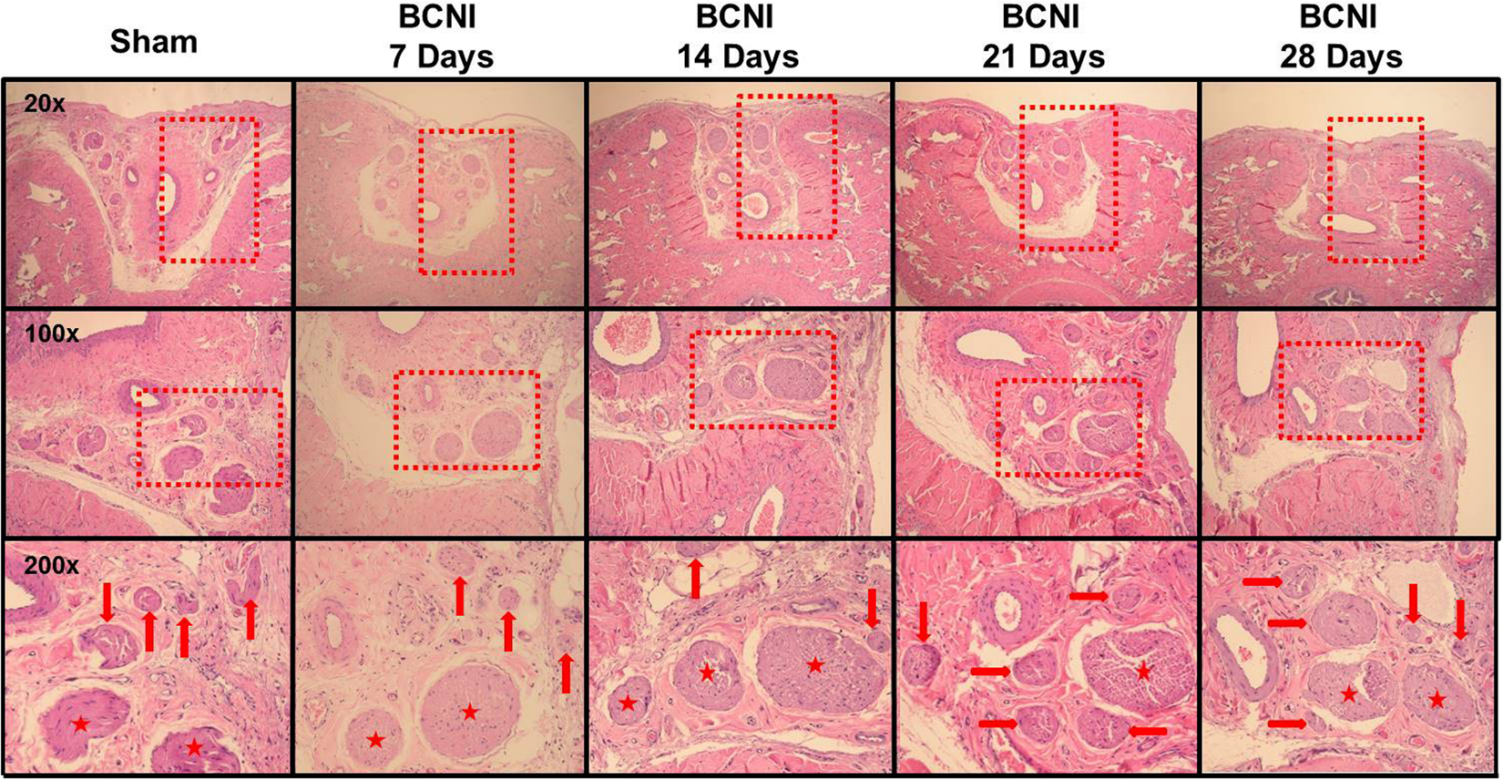
The H&E stain of the dorsal penile nerves’ major and minor branches on different days after bilateral cavernous nerve injury. H&E staining of the dorsal penile nerves’ main and minor branches. The number of small branches was less after bilateral cavernous nerve injury and gradually increased in number subsequently. BCNI = Bilateral cavernous nerve injury. N = 40; Asterisks: dorsal penile nerves’ main branches; Red arrows: dorsal penile nerves’ minor branches.

**Figure 4:**
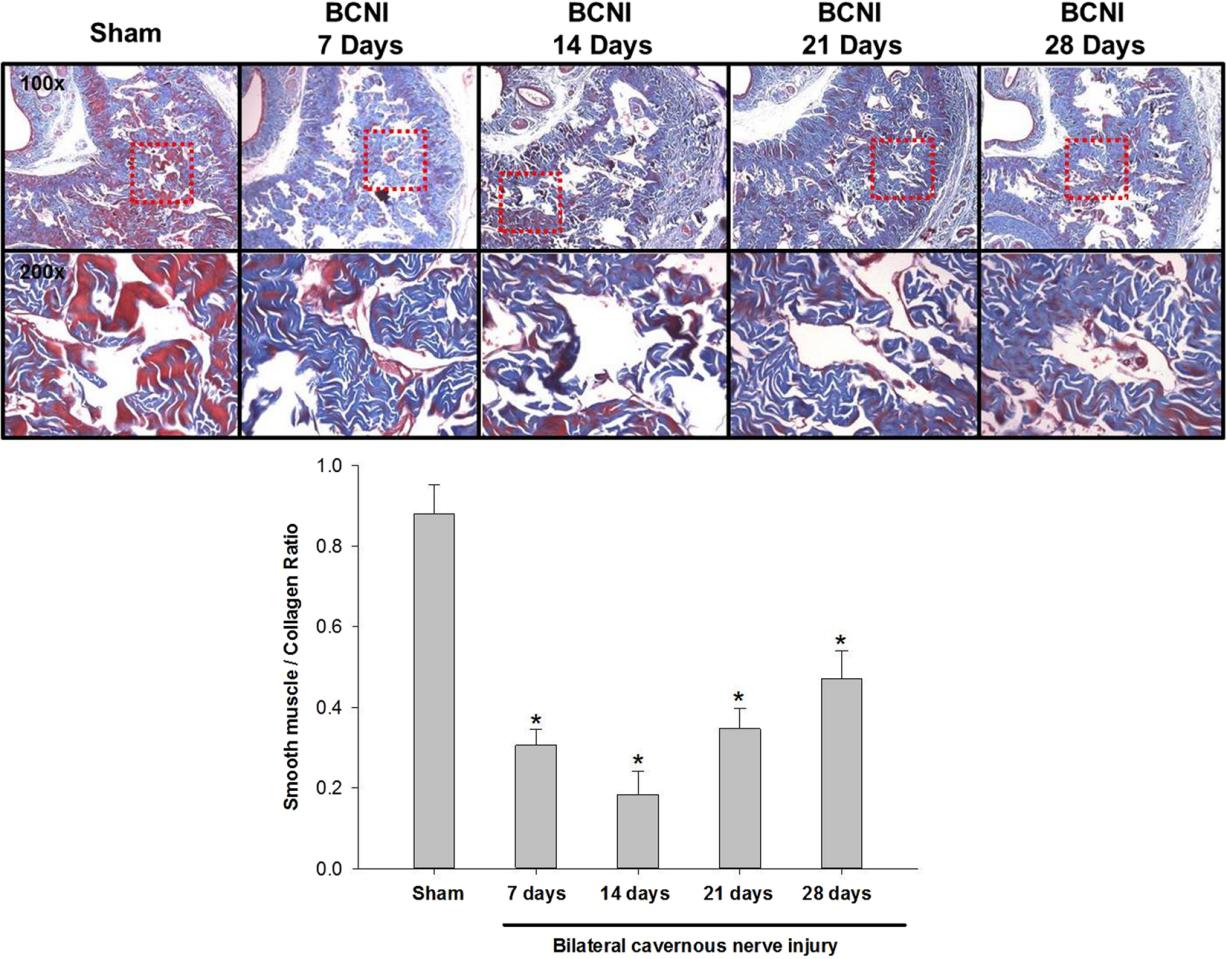
The Masson’s trichrome-stained corpus cavernosum on different days after bilateral cavernous nerve injury. Representative micrographs of Masson’s trichrome-stained rat penile sections. Cavernosal fibrosis was observed in all of the bilateral cavernous nerve injury groups. Smooth muscle was stained red, and collagen fibers were stained purple-blue. BCNI = Bilateral cavernous nerve injury. N = 40; Asterisk: p < 0.05.

**Figure 5:**
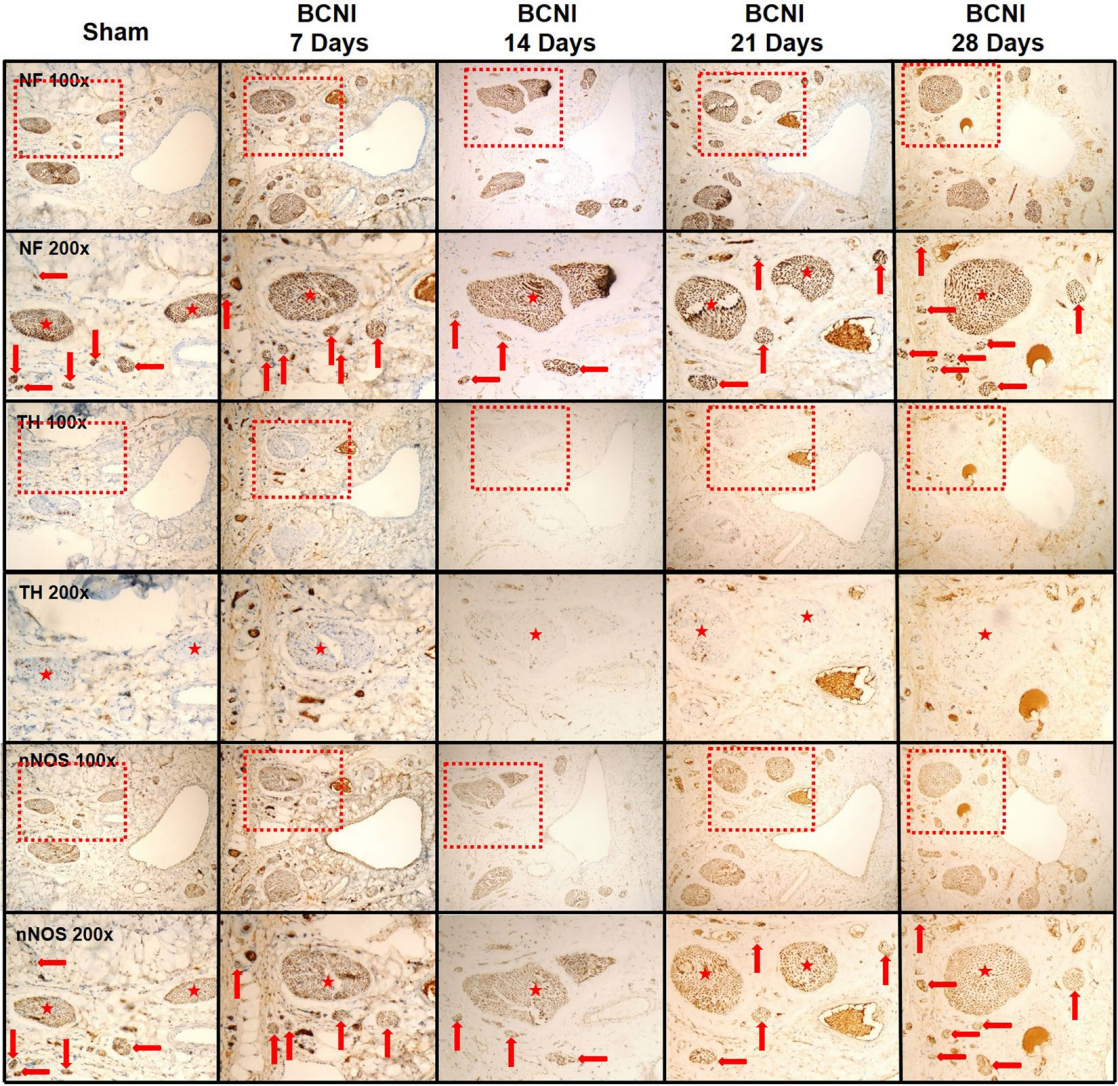
The immunohistochemical stains of the dorsal penile nerves’ major and minor branches on different days after bilateral cavernous nerve injury. To identify the types of nerves, immunohistochemical staining of the neurofilament (pan-nerve marker), tyrosine hydroxylase (sympathetic nerve marker) and neuronal nitric oxide synthases (parasympathetic nerve marker) was performed. Most of the dorsal penile nerves’ minor branches were neuronal nitric oxide synthases positive. However, there were few sympathetic nerve markers of tyrosine hydroxylase expression in either the main or minor branches of the DPNs. BCNI = Bilateral cavernous nerve injury. DPNs = Dorsal penile nerves. Asterisk: dorsal penile nerves’ main branches; Red arrow: dorsal penile nerves’ minor branches.

**Figure 6:**
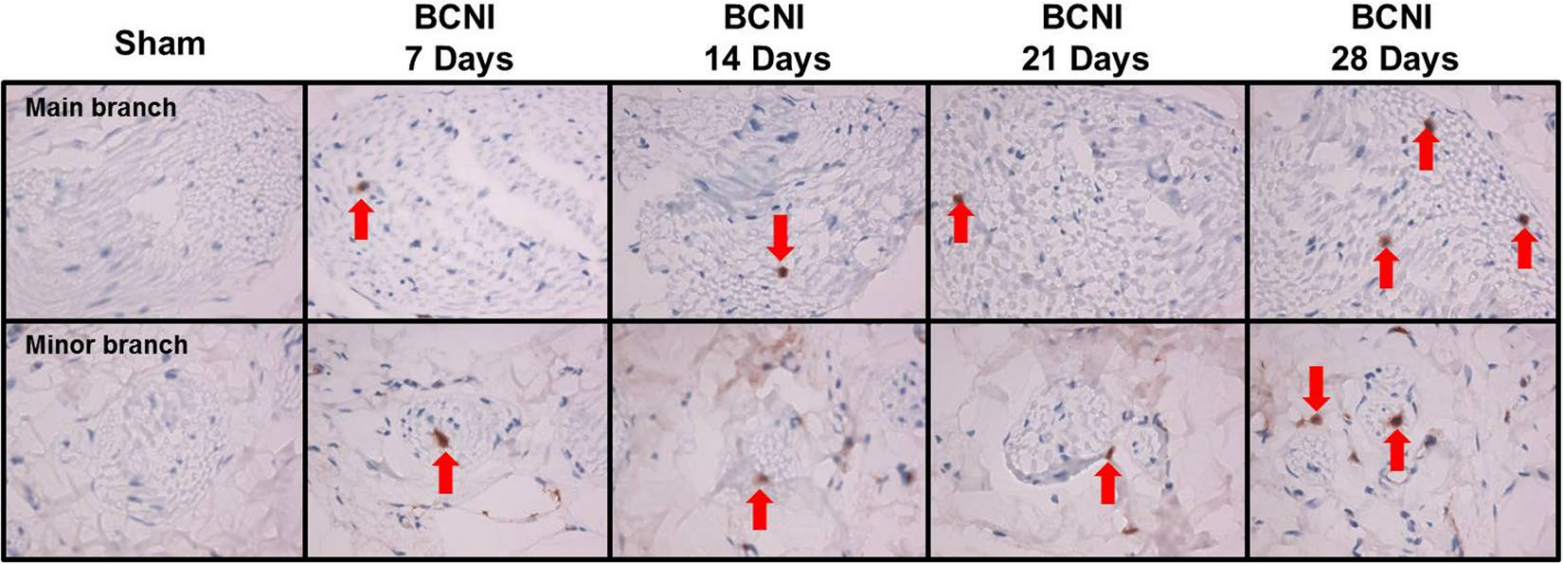
The Ki-67 of the dorsal penile nerves’ major and minor branches on different days after bilateral cavernous nerve injury. Proliferative marker of Ki-67 in the DPNs’ main (upper panels) and minor (lower panels) branches. The positive cells (red arrow) were greater in all of the BCNI groups, compared to the sham group. Moreover, the positive cells were greatest on the 28^th^ day after BCNI. Magnification is 400×. DPNs = Dorsal penile nerves. BCNI = Bilateral cavernous nerve injury.

**Figure 7:**
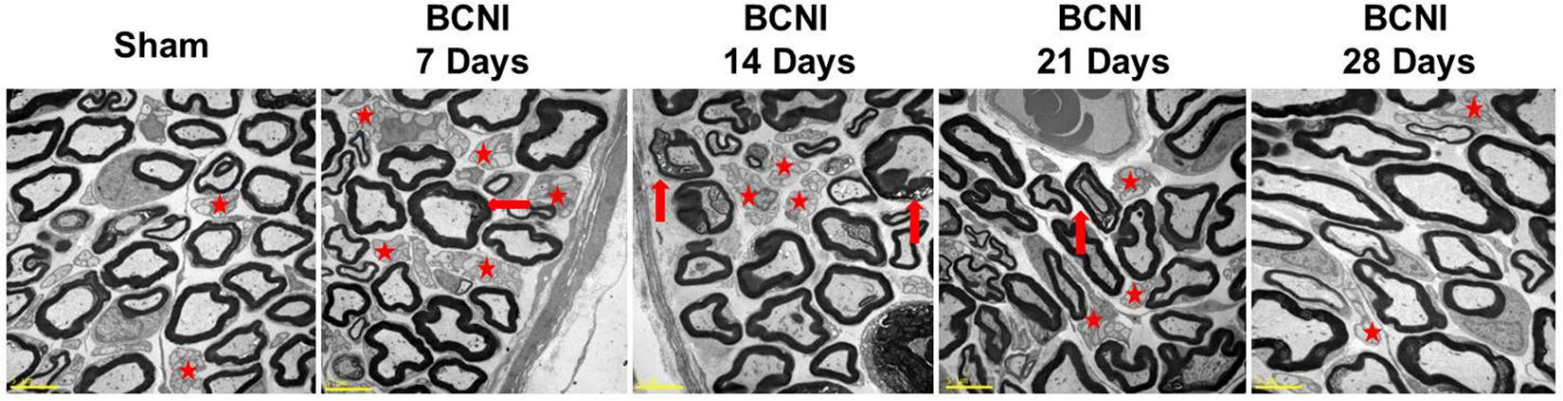
Transmission electron microscope images of changes in the Schwann cells of the dorsal penile nerves’ minor branches. The myelin sheaths of the dorsal penile nerves’ minor branches became loose at the 7^th^ day after bilateral cavernous nerve injury and worsened by the 14^th^ day. Subsequently, the recovery process began, and the myelin sheaths became more compact over the following 14 days (21^th^ day and 28^th^ day after BCNI). BCNI = Bilateral cavernous nerve injury. Asterisk: Unmyelinated nerves; Red arrow: Injured myelin sheaths. 2500× in all pictures.

**Figure 8:**
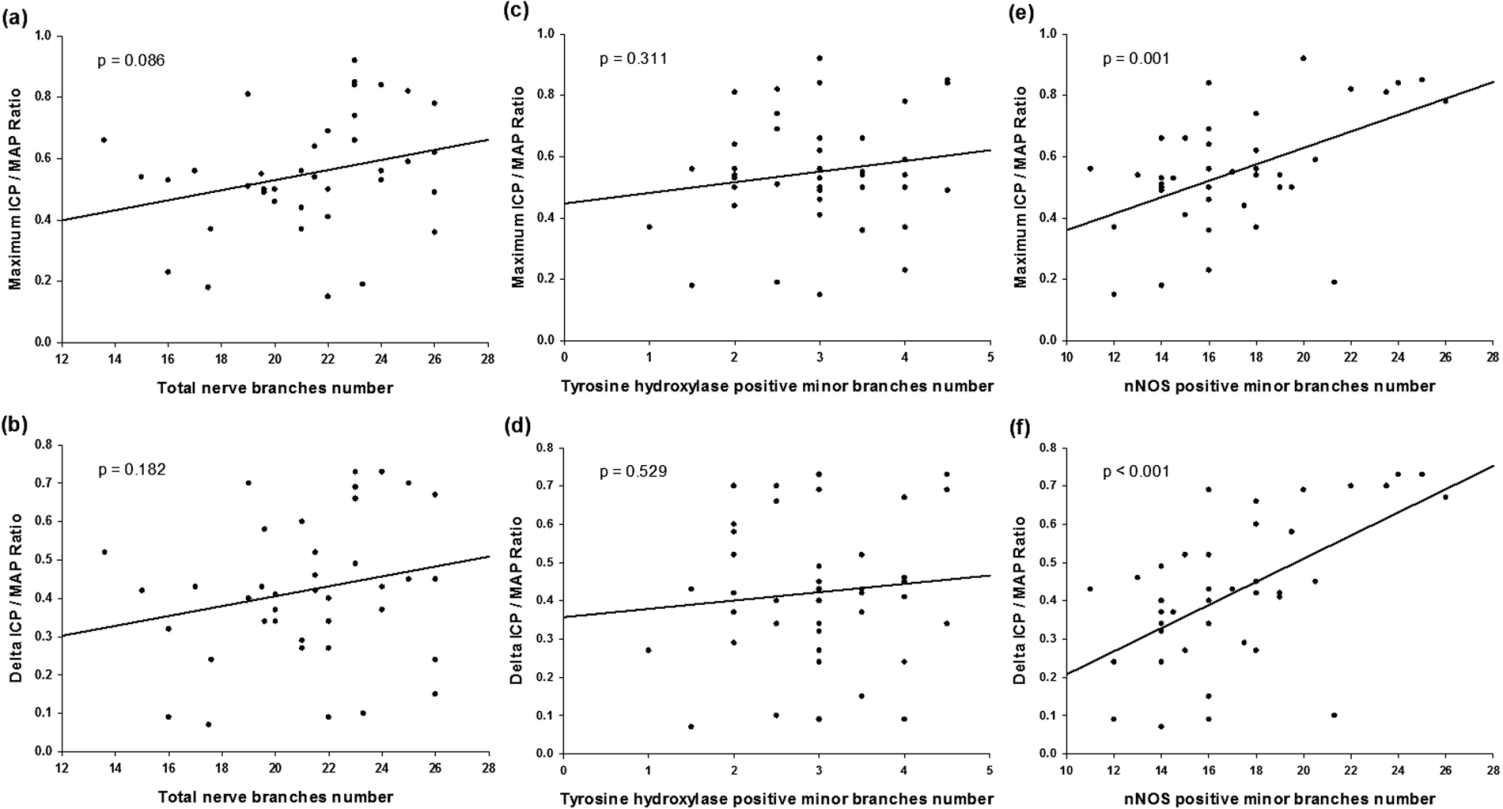
The correlation between the number of dorsal penile nerves’ minor branches and erectile function. The correlation study between the numbers of different nerve fibers and erectile function. The results showed that the number of the nNOS-positive DPN minor branches was positively correlated with the maximum ICP/MAP ratio and with the ∆ICP/MAP ratio of the rats. On the contrary, no statistical correlation was seen in both total nerve branches number and tyrosine hydroxylase positive minor branches. N = 40. nNOS = neuronal nitric oxide synthases. DPNs = Dorsal penile nerves. ICP = Intracavernous pressure. ∆ICP = Changes in ICP. MAP = Mean arterial blood pressure.

Figure 1 shows the anatomical distribution of the major pelvic ganglia, the CNs and the communicating nerve branches to the DPNs in the rats. The DPNs connected to the CNs through the communicating nerve branches. Table 1 provides detailed characteristics of the DPNs with and without BCNI on different days. The DPNs could be divided into main and minor branches. The determined criterion for the main and minor branches of the DPNs were the sizes of the nerve branches. If the nerve branches were smaller than the dorsal artery at the dorsal side of the penis between the tunica albuginea and the Buck’s fascia, it was determined to a minor branch of the DPNs. The main branches of the DPNs were 2-3 in number on each side of the dorsal penis (2–6 in total number) and were located near the dorsal artery. The minor branches of the DPNs, with a mean number of 24.1, were observed and located around the main branches. All of the characteristics of the main branches of the DPNs, including the number of branches, size of the branches, neurite counts per branch and number of blood vessels within the branches, showed no differences between the sham group and the BCNI groups. Similar findings were also observed in the minor branches of the DPNs, except for their number. There were fewer minor branches of the DPNs in the BCNI groups for the first 3 weeks, with a p value of 0.042. According to the time course of erectile function on different days after BCNI (Fig. 2), the erectile function was the worst on the 14^th^ day after BCNI. Subsequently, the erectile function gradually increased over the following 14 days. The number of minor branches of the DPNs was also less in the first 3 weeks after BCNI and gradually increased subsequently. Figure 3 shows representative pictures of the corpus cavernosum. Cavernosal fibrosis was observed in all of the BCNI groups, compared to the sham group (Fig. 4). All of the minor branches of the DPNs were highlighted by the neurofilament stain (Fig. 5). In addition, the majority of these minor branches of the DPNs were demonstrated to be nNOS-positive nerve fibers. However, there were few sympathetic nerve markers of tyrosine hydroxylase expression in either the main or minor branches of the DPNs. Proliferative markers of Ki-67 were observed in both the major and minor branches of the DPNs after BCNI (Fig. 6). TEM also supported the evidence that the minor branches of the DPNs sustained the worst damage to the myelin sheaths on the 14^th^ day after BCNI and partially recovered the myelin sheaths subsequently (Fig. 7). We also performed a correlation study between the numbers of different nerve fibers and erectile function. The results showed that the number of the nNOS-positive DPN minor branches was positively correlated with the maximum ICP/MAP ratio and with the ∆ICP/MAP ratio of the rats (Fig. 8). When we sub-analysis for the number of the DPN minor branches (Supp Table 1), the number of nNOS-positive DPN minor branches were also correlated with the erectile function in the sham group, 7^th^ day, 14^th^ day, 21^th^ day and 28^th^ day after BCNI.

**Table 1:** Characteristics of the dorsal penile nerve in the sham and bilateral cavernous nerve injury groups.

	Sham	BCNI (days)				p value
		7	14	21	28	
N	8	8	8	8	8	—
Main branches						
Number of branches	3.55 ± 0.93	3.51 ± 1.20	3.52 ± 0.93	3.88 ± 0.83	3.84 ± 0.64	0.809
Size of the branches (×10^−2^ mm^2^)	4.19 ± 0.51	4.24 ± 0.54	4.28 ± 0.55	4.13 ± 0.36	4.04 ± 0.50	0.885
Neurite counts per branch	360.75 ± 25.81	347.13 ± 20.47	357.88 ± 26.84	356.13 ± 28.64	354.75 ± 18.05	0.840
Number of blood vessels within the branches	1.28 ± 0.14	1.35 ± 0.36	1.41 ± 0.22	1.31 ± 0.24	1.31 ± 0.21	0.843
Minor branches						
Number of branches	24.13 ± 1.36	20.66 ± 3.60	20.06 ± 4.20	20.00 ± 2.31	21.30 ± 1.99	0.042
Size of the branches (×10^−2^ mm^2^)	1.76 ± 0.22	1.61 ± 0.33	1.64 ± 0.25	1.51 ± 0.20	1.73 ± 0.32	0.393
Neurite counts per branch	26.71 ± 2.32	24.83 ± 2.37	25.83 ± 1.93	24.88 ± 2.97	26.79 ± 1.60	0.261
Number of blood vessels within the branches	0.078 ± 0.035	0.099 ± 0.039	0.085 ± 0.048	0.096 ± 0.050	0.096 ± 0.049	0.868

BCNI = bilateral cavernous nerve injury. All of the data are shown as the mean ± SD.

## Discussion

Our study demonstrated the existence of communicating nerve branches between the DPNs and CNs in rats, similar to previous reports found in human adult and infant cadavers^30–34^. In this study, erectile function was observed to be the worst on the 14^th^ day after BCNI, and it partially recovered 28 days after injury (Fig. 2). The number of nNOS-positive minor branches of the DPNs was positively correlated with erectile function (Fig. 8). Injury of the CNs affected the number of nNOS-positive major and minor branches of the DPNs through communicating nerve branches. When erectile function partially recovered 28 days after BCNI, the number of small branches of the DPNs was also increased. Damage to the small branches of the DPNs was further confirmed by TEM with similar progression (Fig. 7). The damage to the myelin sheaths of the small branches of the DPNs was the worst on the 14^th^ day after BCNI, and it partially recovered at 28 days after injury. Proliferative markers for Ki-67 provided indirect evidence of partial recovery of the major and minor branches of the DPNs. The loss of the minor branches of the DPNs could be a representative feature of BCNI to the DPNs when examined using a light microscope.

Although pathological changes in the cavernosum have been reported in several studies, no studies have reported morphological changes of the DPNs after BCNI. The morphological changes of the main branches of the DPNs were observed to be very mild, with almost no significant changes on the H&E slides in our study. However, the loss of the minor branches of the DPNs is remarkable. These minor branches surround the main branches and have never been mentioned before in the literature. The number of minor branches of the DPNs decreased in the first three weeks after BCNI and gradually increased afterward. Nevertheless, on the 28^th^ day after BCNI, the number of minor branches of the DPNs was still lower than that in the sham group. The greatest damage and lowest erectile function were also seen in the 14^th^ day after BCNI. However, the findings in the main DPNs were reported previously and also in our previous study (Data not shown). These changes included loss of nNOS and increased apoptosis and fibrosis after BCNI^22–26^. Nevertheless, the morphological finding in the DPNs was barely not seen in the light microscope but can be seen the nerve damage changes in the TEM. The current study demonstrated that the minor branches of the DPNs also play an important role in the erectile function. The minor branches should come from the main DPN nerves and sprout out when the DPN goes toward the glans of penis. We found that these DPN minor branches share the similar pathological changes with the DPN main branches. However, the main difference between the main and minor branches were the diminishing in number of branches. Because these DPN minor branches were too small and the injury would make them disappear in the microscope. Moreover, the regeneration of the DPN would be seen in these DPN minor branches by regain the number toward the sham group. On the other hand, the loss of the neurites in the main DPNs were too little to reach the statistical significance. Most of the minor branches of the DPNs were nNOS-positive nerve fibers, which were confirmed by immunohistochemical staining (Fig. 5). Our study also proved that the number of these nNOS-positive nerve fibers was positively correlated with erectile function (Fig. 8). In contrast, a correlation between the total nerve number and the tyrosine hydroxylase-positive nerve (sympathetic nerve) number was not observed, which confirmed that the loss of nNOS-positive minor branches of the DPNs was representative morphological evidence of the rat BCNI model, primarily observed by light microscope (Fig. 3).

The mechanism of the human erection requires the coordination of intact neuronal systems, including the CNs, the DPNs and the perineal nerves. In anesthetized Japanese monkeys, electrical stimulation of the CNs caused increased ICP and penile erection, and atropine enhanced the pressure response^35^. Multiple pathways have been proposed for the course of adrenergic fibers to the penis. Interruption of the sensory branch of the pudendal nerve reduced the adrenergic innervation of cavernosal smooth muscle by 86%^36^. The dramatic reduction of adrenergic innervation of the penis after sectioning of the pudendal nerve, especially the sensory branch, and the absence of neurons in the sympathetic chain suggested that the pudendal nerve is the major pathway by which adrenergic fibers reach the penile tissue in rats^36^. In fetal penile specimens, the continuation of the dorsal neurovascular bundle of the prostate was documented under the pubic arch, where, at the penile hilum, the CNs were found to convey nNOS-positive branches to the DPNs to transform their immunoreactivity to nNOS positive. Interaction between the nNOS-positive DPNs branches and the perineal nerves occurs at the cavernous-spongiosal junction, where the bulbospongiosus muscle terminates^30^. Similarly, the nNOS-negative, ventrally located perineal nerve, originating from the pudendal nerve, becomes nNOS reactive at the cavernous-spongiosal junction^30^. Our results clearly demonstrated that there are communicating nerve branches between the DPNs and the CNs in rats. Most of the communicating nerve branches are nNOS-positive nerve fibers. The continuum of the DPNs and the CNs could be indirectly supported by the clinical assessment of the penile thermal sensory thresholds to reflect the CN damage caused by RP surgery. Penile sensory thresholds for warm and cold sensations significantly increased after non-nerve-sparing RP surgery but not after nerve-sparing RP surgery^37^. Therefore, the CNs travel through the communicating nerve branches to the DPNs and send nNOS-positive fibers to join the DPNs, thereby changing the functional characteristics of the DPNs in both humans and rats.

The regeneration of nNOS-containing nerve fibers is one of the main parameters for the restoration of erectile function. Many studies have reported that different intracavernosal injection materials preserved erectile function after BCNI. However, not all of these injection materials worked through the mechanisms of nerve regeneration. Our laboratory has shown that injection of platelet-rich plasma resulted in better ICP after BCNI^38,39^. The neuroprotective effect of platelet-rich plasma injection into the corpus cavernosum was proved by the increase in the number of myelinated axons, facilitating recovery of erectile function in the BCNI rat model. The effect might be derived from the enriched platelet-derived growth factor in platelet-rich plasma. Previous studies have also demonstrated that growth hormone injection significantly enhanced the regeneration of the nNOS-containing nerve fibers in the DPNs after CN injury^40^. Jung *et al*. revealed that IGF-I and TGF-beta might play key roles in the regeneration of nNOS-containing nerve fibers^41^. In the clinical observation of patients after RP surgery, complete or partial recovery of erectile function never occurred without specific treatment. Therefore, spontaneous regeneration of the injured nerve aroused researchers’ attention. Zhang *et al*. reported on unilateral CN ablation in rats, with nNOS-containing nerve fibers regenerating 6 months later. However, the regeneration of nNOS-containing nerve fibers never occurred in animals with BCNI in their experience^42,43^. Then, 10 years later, Kim *et al*.^44^ found a significant and spontaneous recovery of rats’ erectile function 6 months after BCNI. Our current study further proved this finding at as early as one month. However, the bias of the severity of the crush injury could have caused the recovery time differences between the different studies. In our model, spontaneous nerve recovery could occur as early as 28 days after BCNI by TEM examination. The regeneration process started as early as the 7^th^ day after injury, when the proliferative marker of Ki-67 was observed in either the major or minor branches. The regeneration of the Schwann cell myelin sheaths was especially prominent at the 28^th^ day after BCNI, with more Ki-67-positive Schwann cells. This finding was consistent with the TEM findings observing regeneration on the 28^th^ day. According to the Fig. 8 results, only nNOS positive DPN minor branches’ number was statistical correlated with erectile function. Moreover, the average number of nNOS positive DPN minor branches were statistically different and could be matched with the erectile function (Supp Table 1). Although we have not done any gain / loss function assay, which was really difficult in these animal model, we still suggest that the regeneration of the DPN minor branches would ameliorate erectile function.

## Conclusion

This study demonstrated the gross anatomy of communicating nerve branches between the DPNs and the CNs in rats. Furthermore, the minor branches of the DPNs play an important role associated with erectile function in rats after BCNI. Both the number of and ultrastructural, morphological changes to the minor branches of the DPNs after BCNI were clearly demonstrated using light microscopy and TEM. The loss of the minor branches of the DPNs and the reduced number of nNOS-positive nerves could be a representative feature of the DPNs after BCNI. The number of nNOS-positive minor branches of the DPNs was associated with erectile function after BCNI in rats.

## Electronic supplementary material

Supplementary Information
