# Supplementary material for: nNOS-positive minor-branches of the dorsal penile nerves is associated with erectile function in the bilateral cavernous injury model of rats
Source: Sci Rep. 2018 Jan 17;8:929. doi: 10.1038/s41598-017-18988-2 (PMC5772051; doi:10.1038/s41598-017-18988-2)
Supplement: Supplementary file 1 — Supplementary Information [file 41598_2017_18988_MOESM1_ESM.doc]

**nNOS-positive minor-branches of the dorsal penile nerves is associated with erectile function in the bilateral cavernous injury model of rats**

Yen-Lin Chen^1,2,3^, Ting-Ting Chao^4^, Yi-No Wu^3,5^ , Meng-Chuan Chen^6^, Ying-Hung Lin^3^, Chun-Hou Liao^3,5,7^, Chien-Chih Wu^8,9^, Kuo-Chiang Chen^3,5,10^, [Shang-Shing P. Chou](http://www.mdpi.com/search?authors=Shang-Shing P. Chou)^2^, Han-Sun Chiang^3,8^

Department of Pathology, Cardinal Tien Hospital^1^, Department of Chemistry, Fu-Jen Catholic University^2^, Graduate Institute of Basic Medicine, Fu-Jen Catholic University^3^, Medical Research Center, Cardinal Tien Hospital^4^, Ph. D Program in Nutrition & Food science, Fu Jen Catholic University^5^, Graduate Institute of Medical Sciences, National Defense Medical Center^6^, Division of Urology, Department of Surgery, Cardinal Tien Hospital^7^, Department of Urology, Taipei Medical University Hospital^8^, School of Medicine, Taipei Medical University^9^, Department of Urology, Cathay General Hospital^10^, Taipei, Taiwan

Address correspondence to:

Han-Sun Chiang, M.D. PhD.

Graduate Institute of Basic Medicine, Fu-Jen Catholic University

E-mail address: 05382@mail.fju.edu.tw

Tel.: +886 2 29053904; Fax: +886 2 22193506.

Supplement table 1. Detailed characteristic of different type nerve number and erectile function in sham and injury groups

|  | Sham | | | BCNI (days) | | | | | | | | | | | | p value |
| --- | --- | --- | --- | --- | --- | --- | --- | --- | --- | --- | --- | --- | --- | --- | --- | --- |
|  |  |  |  | 7 | | | 14 | | | 21 | | | 28 | | |  |
| n | 8 | | | 8 | | | 8 | | | 8 | | | 8 | | | --- |
| Nerve type number | | | | | | | | | | | | | | | | |
| Neurofilament positive nerves | 24.13 | ± | 1.36 | 20.66 | ± | 3.60 | 20.06 | ± | 4.20 | 20.00 | ± | 2.31 | 21.30 | ± | 1.99 | 0.042 |
| TH positive nerves | 3.38 | ± | 0.83 | 3.31 | ± | 0.84 | 2.88 | ± | 0.92 | 2.81 | ± | 0.59 | 2.44 | ± | 1.05 | 0.192 |
| nNOS positive nerves | 21.13 | ± | 3.68 | 14.31 | ± | 1.39 | 13.88 | ± | 1.73 | 16.56 | ± | 1.64 | 19.48 | ± | 2.31 | < 0.001 |
| Erectile function | | | | | | | | | | | | | | | | |
| Maximum ICP (cm H_2_O) | 133.76 | ± | 5.77 | 77.95 | ± | 11.27 | 51.48 | ± | 20.24 | 74.44 | ± | 8.01 | 90.72 | ± | 32.85 | < 0.001 |
| Minimum ICP (cm H_2_O) | 22.92 | ± | 7.15 | 22.15 | ± | 8.65 | 21.78 | ± | 8.61 | 18.02 | ± | 2.12 | 17.66 | ± | 2.94 | 0.351 |
| Delta ICP (cm H_2_O) | 110.84 | ± | 10.44 | 55.80 | ± | 12.83 | 29.70 | ± | 17.75 | 56.42 | ± | 9.79 | 73.09 | ± | 32.08 | < 0.001 |
| Area under curve | 5863.87 | ± | 1092.74 | 2320.91 | ± | 673.67 | 1282.41 | ± | 910.30 | 2030.01 | ± | 466.80 | 2464.54 | ± | 901.67 | < 0.001 |
| Mean arterial pressure (cm H_2_O)) | 168.63 | ± | 18.15 | 141.00 | ± | 21.62 | 142.67 | ± | 19.80 | 151.06 | ± | 4.38 | 164.21 | ± | 18.83 | 0.009 |
| Maximum ICP / MAP ratio | 0.80 | ± | 0.09 | 0.56 | ± | 0.11 | 0.37 | ± | 0.17 | 0.49 | ± | 0.05 | 0.52 | ± | 0.19 | < 0.001 |
| Delta ICP / MAP ratio | 0.66 | ± | 0.09 | 0.40 | ± | 0.09 | 0.22 | ± | 0.14 | 0.37 | ± | 0.06 | 0.45 | ± | 0.20 | < 0.001 |

BCNI = bilateral cavernous nerve injury; TH = tyrosine hydroxylase; nNOS = Neuronal nitric oxide synthases; ICP = intracavernous pressure; MAP = Mean arterial pressure.

The mean weight of male rat in the 12^th^, 13^th^, 14^th^, 15^th^, 16^th^ week was 446.9 g, 479.3 g, 491.9 g, 508.8 g and 509.4 g.

All data was showed in mean ± SD.
